# Quantitative Study of Elasticity of Rabbit VX_2_ Liver Tumor with Alternated Cooling and Heating Treatment based on ARFI Ultrasound Imaging Technique

**DOI:** 10.1038/srep29303

**Published:** 2016-07-06

**Authors:** Di Sun, Cong Wei, E. Shen, Tao Ying, Bing Hu

**Affiliations:** 1 Department of Ultrasound in Medicine, Shanghai Jiao tong University Affiliated Sixth People’s Hospital, Shanghai, China; 2 Shanghai Institute of Ultrasound in Medicine, Shanghai, China; 3 Medical Imaging Institute of Shanghai Jiao tong University, Shanghai, China

## Abstract

Acoustic radiation force impulse (ARFI) ultrasound imaging technique is used to quantitatively evaluate the elasticity of rabbit VX_2_ liver tumor with alternated cooling and heating treatment (ACHT). ACHT was performed on fifteen VX_2_ liver tumor models established in fifteen male New Zealand white rabbits with open tumor plant. ARFI was performed on day 0, 1, 7 and 14 after ACHT and shear wave velocity (SWV) in ARFI was recorded to evaluate the elasticity of the treated area. The SWV value of the lesion on day 0, 1, 7 and 14 was 2.33 ± 0.19 m/s, 3.09 ± 0.40 m/s, 2.64 ± 0.37 m/s and 2.26 ± 0.24 m/s, respectively, indicating the treated areas get stiffer on day 1 and then get softer gradually by day. All the difference between adjacent time points was statistically significant. The SWV value of different parts on day 7 approved that the hardness of the treated area is heterogenous: the treated area in the center >the peripheral strip-shaped area >normal liver tissues, consistent with pathological changes. Meanwhile, ARFI combined with conventional US imaging can qualitatively and quantitatively exam the healing process of rabbit VX_2_ liver tumor after ACHT, and corresponds well to the pathological results.

Nowadays, due to its low cost and noninvasiveness, ultrasound (US) imaging has become one of the most popular imaging method for the identification of solid tumors. However, poor reproducibility and high operator dependence limited the diagnostic value of conventional US^1^. Recently, acoustic radiation force impulse (ARFI) US imaging technique, including virtual touch tissue imaging (VTI) and virtual touch tissue quantification (VTQ) function, has been recognized to be useful^2^,^3^. Based on the principle that localized tissue displacement is less in harder tissues than in softer ones, ARFI can be used to evaluate the tissue stiffness qualitatively and quantitatively, especially for the evaluation of the lesion elasticity of tumors.

Meanwhile, invasive treatment technology is becoming more and more universal concern of human beings. It is well known that the effect of traditional cancer treatments, including surgical resection, radiation and chemotherapy, is limited mainly by metastasis recurrence and destruction of normal organs^4^. In this context, alternated cooling and heating treatment (ACHT), which is applied through energy intervention combining freezing and heating into malignant tissues, has recently been accepted as a promising cancer treatment technique because of its less invasiveness and fewer side effects. In this synergetic process, freezing treatment damages the tumor cells by the strategies of direct cellular injury, micro-vascular damage and possible immunologic responses^5^. Compared with high temperatures (above 60 °C) used on Radiofrequency Ablation and High Intensity Focused Ultrasound^6^,^7^ or laser heating which leads to completely necrosis of tumor tissues, mildly heating treatment on 40–45 °C has become the mainstream choice in ACHT for the effectiveness of boosting anti-tumor inflammatory reaction and immune responses^8^. Therefore, hyperthermia (42–43 °C) and freezing was combined to induce immunological response of the subject which could afterwards prevent recurrence and metastasis. Repasky^9^ and Lee^10^ carried out experiments and ACHT has been proven to have the potential to promote systemic anti-tumor immune mechanisms, which may target not only locally surviving tumor cells, but also distant out-of-field metastases^11^.

However, the research of imaging evaluation of in-vivo inflammatory response originating from ACHT was very limited, especially for the quantitative imaging study. To the best of our knowledge, there are no reports on the therapeutic immune response evaluation of ACHT using elastic ultrasonic imaging technology.

In the present work, the novel ARFI imaging technique, including VTI and VTQ function, has been first introduced to evaluate the short-term changes of the lesion elasticity quantitatively before and after ACHT treatment. Conventional 2D US imaging supplies the sampling location guidance for ARFI and color Doppler flow imaging (CDFI) provides blood supply information. The SWV values of different parts (central part, peripheral part) of the lesion and normal liver tissues were also recorded and discussed. The quantitatively elasticity changes of tumor was also examined in contrast to pathological results.

## Results

### Comparison of lesion elasticity by ARFI after ACHT

To clarify the elasticity change of the lesion, comparative SWV values by ARFI at different time points (day0, day1, day7 and day14) and SWVs on day 7 in different lesion areas are shown in Table 1. Clearly, the SWV value of tumors before ACHT is significantly higher than normal liver tissues (P < 0.001). After ACHT treatment, the SWV value of the treated tumor on day 1 gets higher than that of day 0 (P = 0.001). While the SWV on day 7 is significantly lower than that of day 1 (P = 0.011), and day 14 shows a further decrease compared to day 7 (P = 0.028). The results mean the treated areas get stiffer on day 1 after ACHT treatment, and then get softer gradually by day. All the difference between adjacent time points was statistically significant. The SWV value of different parts (central part, peripheral part) of the lesion and normal liver tissues on day 7 was 2.66 ± 0.33 m/s, 2.22 ± 0.39 m/s, 1.09 ± 0.10 m/s, respectively.

### Day 0

**Figure 1:**
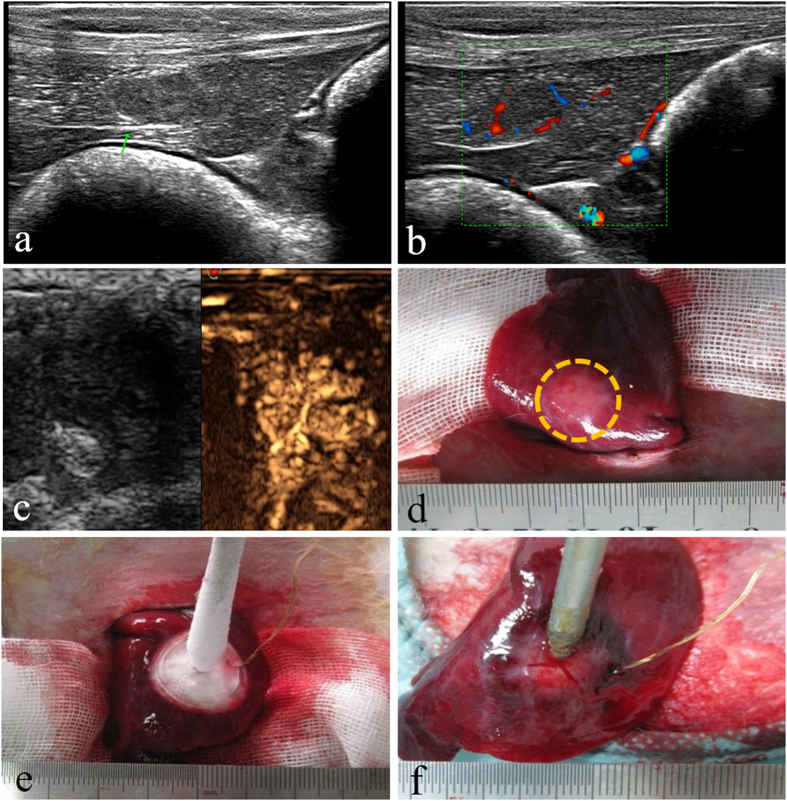
US images before tumor treatment and direct vision during ACHT, (**a,b,c**). 2D, CDFI and CEUS images of tumor on day 0, appearing as a hypervascular round lesion; (**d**). the tumor under direct vision; (**e,f**) cooling and heating treatment separately.

**Figure 2:**
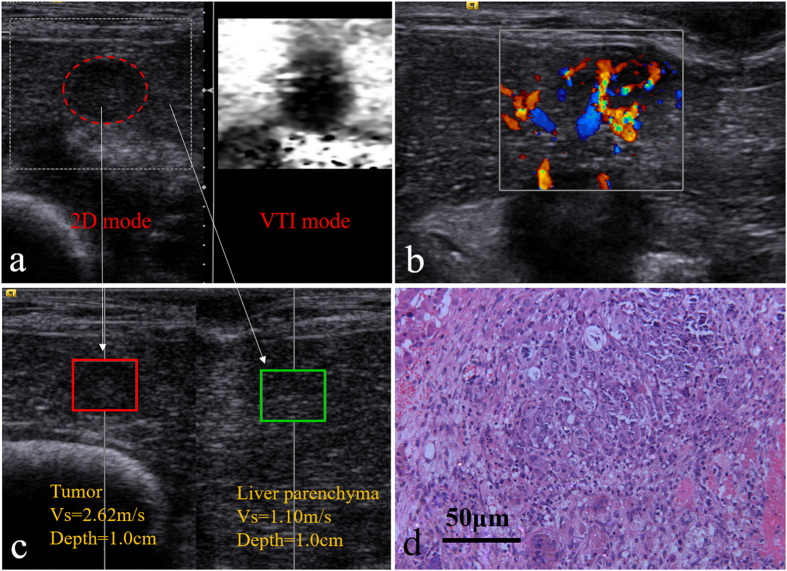
ARFI and pathologic image on day 0, (**a**) the comparative US image of the tumor under VTI mode; (**b**) the CDFI image of tumor; (**c**) the SWV of the tumor and the liver parenchyma under VTQ mode; (**d**) histopathologic picture of the tumor (HE staining, ×200 ).

The US images before tumor treatment and direct vision during ACHT was shown in Fig. 1. Before ACHT treatment, the tumor is well defined on US image. It appears on VTI mode US image as a round-like dark area, indicating the hard nature (Fig. 2a). According to the CDFI image (Fig. 2b), it should be noticed that the tumor was hypervascular compared to the normal liver tissue. The lesion vascularity on day 0 can be identified as grade III in Adler grading system. Clearly, the SWVs are significantly different between tumors and normal liver tissues (2.33 ± 0.19 m/s vs. 1.23 ± 0.10 m/s, Fig. 2c, P < 0.001). The SWV of tumors are much higher than that of normal liver tissues. Pathological result (Fig. 2d) on day 0 indicates the VX_2_ tumor is composed of abundant cancer cells with atypia nuclear, frequent mitoses and hemorrhage.

### Day 1

**Figure 3:**
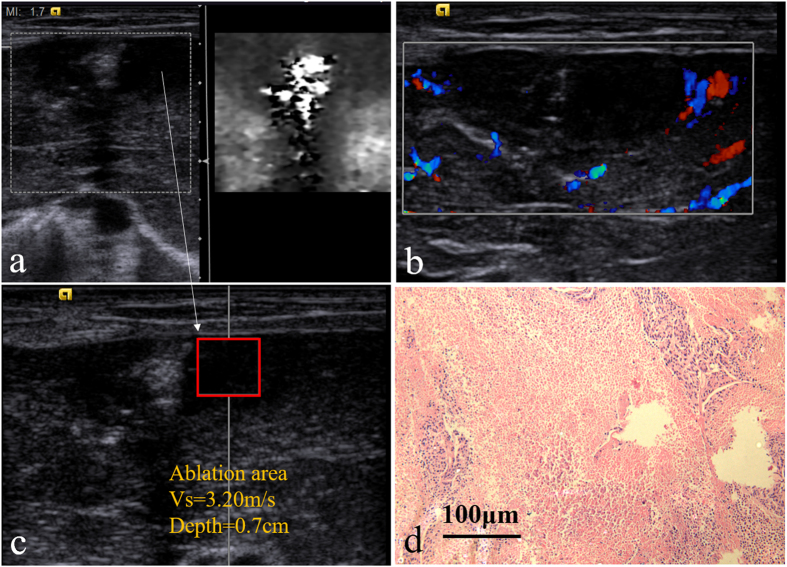
ARFI and pathologic image on day 1, (**a**) the comparative US image of the ablation area under VTI mode; (**b**) image of the ablation area by CDFI; (**c**) the SWV of the ACHT area under VTQ mode; (**d**) histopathologic picture of the tumor (HE staining, ×100 ).

On day 1, the boundary of the treated tumor gets fuzzy on 2D image. On VTI image, the treated area is still dark with poorly-defined boundary (Fig. 3a). The bright region near the tumor on VTI image can be ascribed to the hole caused by the puncture with the ACHT probe. According to the CDFI image (Fig. 3b), there is no visible blood flow within the tumor, while peripheral flow activated to some extent. The lesion vascularity on day 1 can be described as grade 0. The SWV of the lesion on day 1 is 3.09 ± 0.40 m/s (Fig. 3c), which is significantly higher than that of day 0 (P = 0.001). Pathological image on day 1 of the treated lesion shows large-area coagulation necrosis, vascular rupture, and nucleolus fragmentation (Fig. 3d).

### Day 7

**Figure 4:**
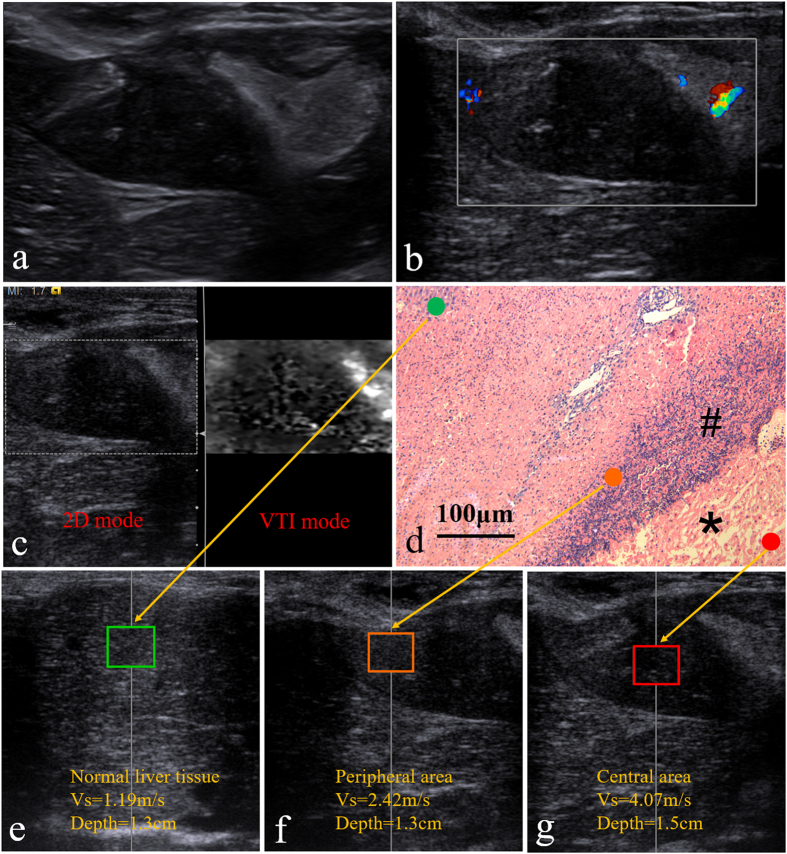
ARFI and pathologic image on day 7, (**a**) 2D US image of the ablation area (**b**) image of the ablation area by CDFI; (**c**) the comparative US image of different areas under VTI mode; (**d**) pathology (HE staining, ×100): necrotic tissues inside ablated lesions (*) and peripheral inflammatory reaction strips (#); (**e,f,g**) SWVs in normal liver parenchyma, peripheral area and central part comparatively under VTQ mode.

On day 7, the boundary of the lesion is not well-defined on 2D image (Fig. 4a) and VTI image. On the CDFI image, there is no visible internal flow with rare peripheral flow (Fig. 4b). The lesion vascularity on day 7 remains grade 0. According to the greyscale value on VTI image (Fig. 4c), the ablated lesion can be divided into two different parts, the central part and the peripheral part. Different from the bright normal liver tissue, the ablated lesion appears dark, and the central part is darker than the peripheral part. These ARFI results indicate the central part of the ablated area is the hardest, followed by peripheral area and normal liver tissues. The SWV of the lesion on day 7 is 2.64 ± 0.37 m/s , which is significantly lower than that of day 1 (P = 0.011).

To further understand the transfer property inflammatory response originating from ACHT, the SWV value of different parts (central part, peripheral part) of the lesion and normal liver tissues on day 7 was also recorded. The hardness or SWV is distributed as follows (Table 1, Fig. 4e–g): central part (2.66 ± 0.3 m/s, Fig. 4g) >peripheral part (2.22 ± 0.39 m/s, Fig. 4f) >normal liver tissues (1.09 ± 0.10 m/s, Fig. 4e), showing significant differences (P < 0.001). Paired comparison shows that SWVs are significantly larger in central part versus peripheral part (P=0.008) and in peripheral part versus normal liver tissues (P < 0.001). Histopathologic picture after HE staining of different lesion areas on day 7 was shown in Fig. 4d. Inflammatory reaction strips appear around the coagulation necrosis, as well as cell injuries, proliferation of lymphocyte, and formation of granulation tissues.

### Day 14

**Figure 5:**
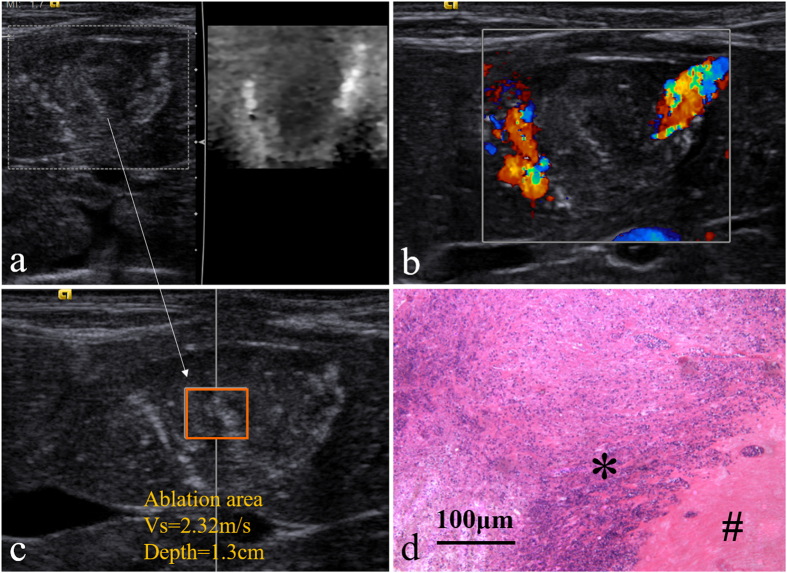
ARFI and pathologic image on day 14, (**a,c**) US images of the ablation area under VTI and VTQ mode; (**b**) the CDFI image of the ablation area; (**d**) histopathologic image of the ablation area: inflammatory cell area (*) and coagulation necrosis area (#) (HE staining, ×100 ).

On day 14, the boundary of the lesion is obscure on 2D image and VTI image. The dark treated lesion on VTI image (Fig. 5a) indicates its hard nature. According to the CDFI image (Fig. 5b) of lesion on day 14, it can be clearly observed that the blood flow enriched from the surrounding to the central part, indicating new vessels developed from the peripheral part to the central part on day 14. The lesion vascularity on day 14 is identified as grade II in Adler grading system. The SWV of the lesion on day 14 is 2.26 ± 0.24 m/s , which shows a further decrease compared to the SWV on day 7 (P = 0.028, Fig. 5c). According to the Pathological picture (Fig. 5d), the margins of inflammatory reaction on the peripheral area extend towards the interior, with lymphocytes, new blood vessels granulation tissues, and a small amount of fiber streaks around.

### Summary

#### Elasticity evaluation

**Figure 6:**
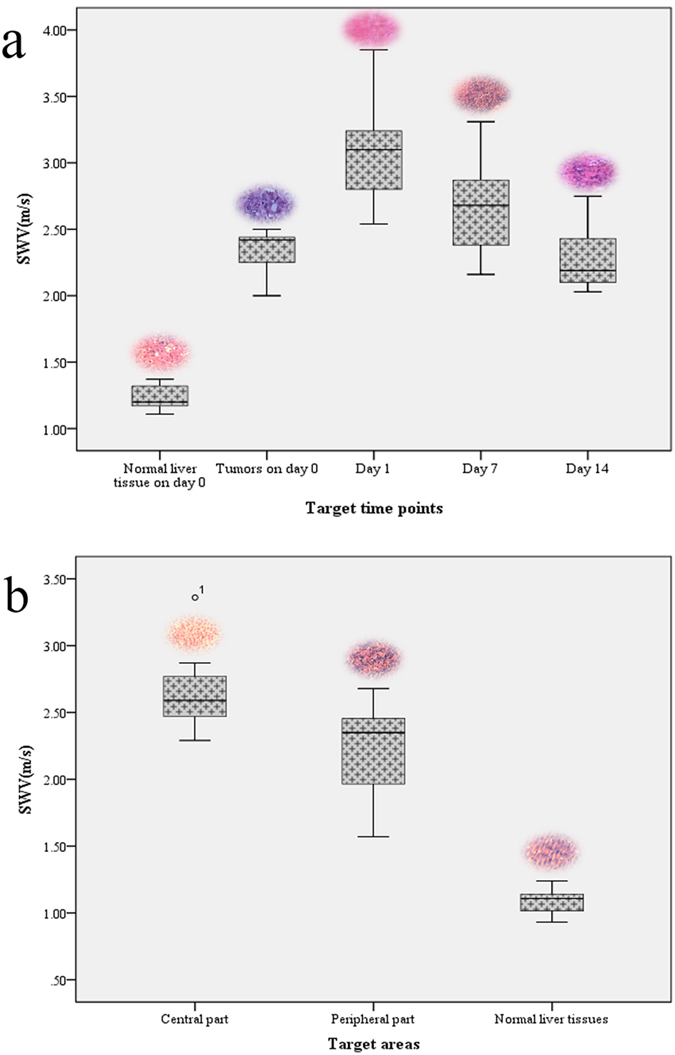
(**a**) SWVs in lesions at different time points after ACHT, (**b**) SWVs in areas with different hardness on day 7 after ACHT (^o^ indicates abnormal values). Inserted are the corresponding pathological images after HE staining.

The treated area on day 1 is significantly harder than on day 0. Then the lesion gets softer continuously on day 7 and 14 (Fig. 6a). On day 7, the hardness of the treated area is heterogenous: the treated area in the center >the peripheral strip-shaped area >the normal liver tissues, consistent with pathological changes (Fig. 6b).

#### Vascularity

The vascularity of the lesion have been quantified by Adler grading system to clarify the flow feature, and lesion flow is classified as grade III, 0, 0 and II on day 0, 1, 7 and 14, respectively. The lesion blood flow disappeared on day 1 and subsequently new vessels developed from the peripheral part to the central part.

#### Pathology

Large-area coagulation necrosis replaced abundant cancer cells on day 1 after ACHT. Subsequently, inflammatory reaction strips appear around the coagulation necrosis on day 7, the margins of inflammatory reaction extend to the interior on day 14.These results can well explain the changes of the elasticity and vascularity of the lesion after ACHT.

## Discussion

The suggestion of ACHT was first put forward by Gage *et al.*^12^ in 1982 and afterwards was used in tumor treatment. Its therapeutic effect is supported by studies at cell level and *in-vivo* animal level^13^.Recently, accumulating evidence indicates that ACHT is promising in minimally invasive therapy on tumor, especially cancer recurrence and metastases^9^,^10^. However, the research on imaging evaluation of inflammatory response originating from ACHT was limited, especially for the quantitative elasticity study.

In this regard, ARFI, as a novel elastic ultrasonic imaging technology combining qualitative and quantitative functions, is especially suitable for elastic measurement of deep organs and widely used in grading of liver fibrosis^14^. There are a few studies about the ARFI-based treatment of solid tumors, such as liver, kidney and breast tumors. There is rare research about tumor ACHT by ARFI. While the evaluations of lesion hardness changes after radiofrequency ablation and the elastography-based determination of treated borders have been reported. As reported, the area and volume of radiofrequency lesions measured by elastic method are both significantly correlated with gross specimens, and it is an effective method to estimate the treatment region^15^. The lesion boundaries on VTI image before and after radiofrequency are clearer than on 2D ultrasound, indicating VTI is probably feasible for treatment guidance^16^.

In this work, ARFI including VTI and VTQ technique was used to observe the short-term dynamic change of lesion hardness after ACHT quantitatively for the first time. Results show the tumor on day 1 after operation is harder than on day 0, and gets softer on day 7, even softer on day 14. The central parts on day 7 are harder than the peripheral parts, as confirmed by VTQ quantitative examination. To avoid impact of depth on the areas of interest, we unified the measurement depth at 1.3–1.5 cm in all areas before and after treatment. With Adler grading system of vascularity, The flow of the lesion was classified to be grade III, 0, 0 and II on day 0, 1, 7 and 14, respectively. The rich flow within the tumor disappeared on day 1 and day 7, and subsequently new vessels developed from the peripheral part towards the central part on day 14.

The results of lesion hardness from ARFI and vascularity of CDFI are consistent with the postoperative HE staining: On day 1, coagulation necrosis occurs in the central parts of ablated lesions, with nucleolus damage, vascular rupture and hard nature. On day 7, strip-shaped inflammatory reaction areas appear in the peripheral parts, showing cell injuries, infiltration by neutrophils, formation of granulation tissues, with hardness in between coagulation necrosis areas and normal liver tissues. On day 14, the inflammatory reaction areas extended from the peripheral part to the central part, while the necrotic tissues were found with inflammatory cells, new blood vessels and granulation tissues, thus becoming softer. It is indicated that ARFI can reflect the pathological changes after treatment, and may become a new elasticity-based method for evaluation of ablation therapeutic effect, in addition to traditional imaging methods. This can well explain the therapeutic process of ACHT on rabbit VX_2_ liver tumor. First, ACHT treatment inactivates tumor cells and destroys its blood supply. Subsequently, inflammatory response is activated and the new vessels develop from the peripheral part towards the central part. All the ARFI results combine with blood supply and pathological results approved ACHT to be a mild and controllable therapeutic method on VX_2_ liver tumor.

So far, the pathology of ACHT is rarely known. Since physical therapies including radiofrequency, induction heating, and surface heat treatment induce similar pathological changes^17^, we deduce ACHT can be explained similarly. As reported, inflammatory reaction was evoked^18^ and maximized on day 3 after radiofrequency ablation, then gradually degraded, and at week 2, turned to fibrosis streaks. In our study, significant inflammatory reaction was also observed on day 7 and 14. After radiofrequency, the lesions were manifested from center to peripheral part as necrosis, inflammatory reaction and fiber belts, residual tumors, and paraneoplastic liver tissues, which are similar to our findings.

Moreover, in one case of our study, the tumor is similar to normal liver tissues on echoes and thus could not be identified by 2D ultrasonography. With the aid of VTI, we found the ellipse-shaped hard region in the target areas. Furthermore, VTQ quantitative detection was employed to compare the hardness of the region with normal liver tissues, and indicated that the SWV of the target region was higher than the peripheral normal liver tissues. Additionally, contrast-enhanced ultrasonography showed hypertransfusion in this area at early stage and confirmed it as tumor. This case indicates ARFI can supplement 2D ultrasound in imaging-based tumor detection. In this regard, ARFI combined with conventional US imaging can provide qualitative and quantitative information of the healing process of rabbit VX_2_ liver tumor after ACHT, and corresponds well to the inflammatory response reflected by pathological results. This will be helpful for further research on therapeutic evaluation of ACHT.

## Conclusions

ARFI ultrasound imaging and elasticity value examination of therapeutic areas was performed in ACHT treated VX_2_ rabbits liver tumor models. The treated area on day 1 is significantly harder than on day 0. Then the lesion gets softer continuously on day 7 and 14. On day 7, the hardness of the treated area is heterogenous: the treated area in the center >the peripheral strip-shaped area >normal liver tissues, consistent with pathological changes. All the results corresponds well to pathological changes and support the argument that ARFI can qualitatively and quantitatively detect the time-varying elastic changes of tumors after ACHT, as well as the elasticity of treatment region in different areas. Thus, ARFI can be a useful new method for observation after ACHT.

## Materials and Methods

### Ethics statement

This research was conducted in accordance with the guidelines of the National Institutes of Health of China for the care and use of laboratory animals. All the experimental protocols were approved by the local animal ethics committee affiliated to Shanghai sixth People’s Hospital.

### Experimental animals

Fifteen male New Zealand white rabbits (provided by the animal experiment center in our hospital), 4-month-old and weight 2.52 ± 0.40 kg, were fed with 150 g/d ordinary pellet feed in single cages.

### Instruments

The ACHT instrument was developed by Med-X Research Institute, Shanghai Jiao Tong University. This instrument uses the same probe for cooling with liquid nitrogen and heating with radiofrequency. The sketch and actual picture of the prototype ACHT system was shown in the Supplementary Information. It can sense the temperature changes at the tumor margins using a thermocouple, and automatically or manually adjust the amplitude and power on the opening/closing voltages and radiofrequency voltage on the low-temperature electromagnetic valves in the main channel.

#### Ultrasonic instrument

An Acuson S2000 color Doppler ultrasonic diagnostic instrument (SIEMENS, German), equipped with built-in ARFI and a 9L4 probe, and was used (frequency 4.0–9.0 MHz, mechanical index 1.7). The instrument parameters (gain, sampling depth, focus range and dynamic range) were adjusted to acquire satisfactory images and fix the parameter setting.

#### Preparation of animal models

(1) Preparation of tumor tissue blocks: From each tumor-bearing rabbit, fresh VX_2_ tumor tissues were taken out from muscles and cut into 1–2 mm^3^ blocks, which were placed in a small amount of normal saline until used. (2) Planting of liver VX_2_ tumor: The rabbits were anesthetized via ear vein injection of 30 mg/kg pentobarbital. The middle and upper parts of abdomen were depilated using an animal shaving device when the rabbits were fixed on an operating table in the dorsal position. After disinfection with iodophor and draping, a 4 cm cut from the xiphisternum along Hunter’s line downward was opened to expose the liver. The exposed liver lobes were carefully pulled out. A smooth-end eye tweezer was used to puncture the thick parts on the lobes (left medial liver lobes), forming a 0.8 cm tunnel. About 2–3 tumor blocks (each 1–2 mm in diameter) as-prepared were transplanted in each tunnel bottom to the thickest part of the rabbit liver. Then after bleeding was stopped with aseptic gauze under light pressure, the lobes were returned to the abdominal cavity. After the incisions were sutured, 0.1 mIU penicillin was intramuscularly injected immediately. In the following 3 days, the same dose was injected each day.

#### ACHT treatment

On day 18 after tumor plant, tumor growth was observed under ultrasound until the diameter increased to about 0.8–1.0 cm, presenting a hypervascular round lesion (Fig. 1a–c). Under direct vision, the tumors were treated with the ACHT instrument. The steps of laparotomy were the same as “model preparation (2)”. After the lobes were pulled out, the tumors were exposed (Fig. 1d). Together with the anterior and posterior diameters, the needle electrode was inserted into the center of a tumor. The temperature measuring needle was inserted at 1.0 cm away from the needle wall. Then the control system was started. First cooling was conducted: liquid nitrogen was added. When the marginal temperature dropped to 0 °C and after 10 min (Fig. 1e), the liquid nitrogen valve was closed, until the treated area naturally recovered to room temperature. Then the area was heated by electrode radiofrequency to 43 °C and maintained for 20 min (Fig. 1f). After treatment, the needle was taken out. After the treated area naturally recovered to room temperature, the abdomen was closed. The intramuscular injection of penicillin was the same as in “model preparation (2)”.

#### Conventional ultrsonography

The conventional US images were obtained for each target lesion. The lesion was evaluated for size, echogenicity, margin (well or poorly defined), intranodular vascularity on different time(day0, day1, day7 and day14).The classic Adler grading system^19^ was adopted to quantify the vascularity in the lesion compared with normal liver tissue, in which grade 0 means almost no visible flow, and grade 0,I,II,III mean more and more abundant blood supply.

#### ARFI examination

The ARFI instrument built-in Acuson S2000 was used for examinations at 0, 1, 7 and 14 d after operation (marked as day 0, 1, 7 and 14). First, VTI was started to collect grayscale value of elasticity. On this basis, VTQ was used to quantitatively detect hardness in different areas. The size of the color frame is 6 × 5 mm. During measurements, the probe was suspended on the body surfaces (in contact without generating pressure). Each area was detected 5 times, with shear wave velocity (SWV) recorded. The SWV measurement of the whole tumor lesion was independent from the central part and the peripheral part on day 7. According to the Young’s modulus formula blew, the relationship of SWV and 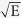 (Young’s modulus, Pa) is proportional:


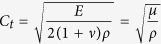


where *Ct* is shear wave velocity; *E* is longitudinal/axial elastic Young’s modulus (modulus) ;*μ* is transverse elastic modulus (shear modulus);*ρ* is tissue density;*ν* is Poisson’s ratio, which is usually 0.499 for soft tissues.

#### Image Interpretation

In VTI images, grayscale is a general measure of elastography, which was classified into two values (represent in black or white color) through the comparison between the lesion and the surrounding liver tissue. Darker region indicated stiffer tissue.

#### Pathological examination

After ARFI was examined on day 0, 1, 7 and 14, one rabbit was sacrificed each time. The lesion and peripheral tissues were cut out, fixated with formalin, and stained with Hematoxylin & Eosin (HE). The amplification of the HE staining photos is ×200 on day 0 and ×100 on day 1, 7 and 14.

### Statistical analysis

Data were analyzed on SPSS 23 (IBM Corporation, Armonk, NY, USA). Measured data were expressed as “mean ± standard deviation”. The ARFI data between normal liver tissues and tumors were compared with independent sample t-test. The data between day 0 and 1, day 1 and 7 were compared by paired t-test, the same as day 7 and 14. The data in different parts on day 7 were tested with analysis of variance (ANOVA), which are the central part, peripheral part of the lesion and the liver tissue respectively. Then data between-groups were tested. Groups meeting variance homogeneity were tested by least significant difference method (LSD). In case of variance unjustification, Dunnett T3 method was used, with significance level at P < 0.05.

## Additional Information

**How to cite this article**: Sun, D. *et al.* Quantitative Study of Elasticity of Rabbit VX_2_ Liver Tumor with Alternated Cooling and Heating Treatment based on ARFI Ultrasound Imaging Technique. *Sci. Rep.* **6**, 29303; doi: 10.1038/srep29303 (2016).

## Supplementary Material

Supplementary Information

## Figures and Tables

**Table 1:** SWVs in lesions at different time points (day0, day1, day7 and day14) and SWVs on day 7 in different lesion areas.

Target	Number of cases	SWV (m/s)*	P	Note of P value
Normal liver tissues on day 0	15	1.23 ± 0.10 (1.15 ~ 1.30)	–	–
Tumors on day 0	15	2.33 ± 0.19 (2.18 ~ 2.47)	<0.001	Tumor-Liver tissue
Day 1	14	3.09 ± 0.40 (2.79 ~ 3.40)	0.001	Day1-Day0
Day 7	13	2.64 ± 0.37 (2.36 ~ 2.93)	0.011	Day7-Day1
Central part(C)	13	2.66 ± 0.33 (2.39 ~ 2.94)	0.008	C-P
Peripheral part(P)	13	2.22 ± 0.39 (1.90 ~ 2.55)	<0.001	P-N
Normal liver tissues(N)	13	1.09 ± 0.10 (1.01 ~ 1.17)	–	–
Day 14	12	2.26 ± 0.24 (2.08 ~ 2.45)	0.028	Day14-Day7

^*^Data are expressed as means ± standard deviations, with 95% confidence interval in parentheses.
