# Supplementary material for: Quantitative Study of Elasticity of Rabbit VX2 Liver Tumor with Alternated Cooling and Heating Treatment based on ARFI Ultrasound Imaging Technique
Source: Sci Rep. 2016 Jul 6;6:29303. doi: 10.1038/srep29303 (PMC4933955; doi:10.1038/srep29303)
Supplement: Supplementary Information [file srep29303-s1.doc]

Supplementary Information for

Quantitative Study of Elasticity of Rabbit VX_2_ Liver Tumor with Alternated Cooling and Heating Treatment based on

ARFI Ultrasound Imaging Technique

**Di Sun**^1,2^†**, Cong Wei**^1,2^†**, E Shen**^2^***, Tao Ying**^1, 3^***, Bing Hu**^1,2^*****

^1^Department of Ultrasound in Medicine, Shanghai Jiao tong University Affiliated Sixth People's Hospital

^2^Shanghai Institute of Ultrasound in Medicine

^3^Medical Imaging Institute of Shanghai Jiao tong University

† *These authors contributed equally to this work.*

** Correspondence and requests for materials should be addressed to B.H. (email:* [*binghu_zz@163.com*](mailto:binghu_zz@163.com)*).*


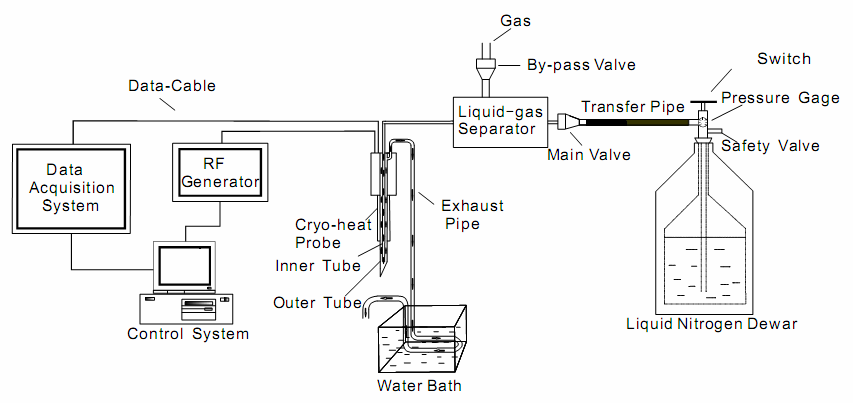


Fig.S1 Sketch of the ACHT Instruments system.


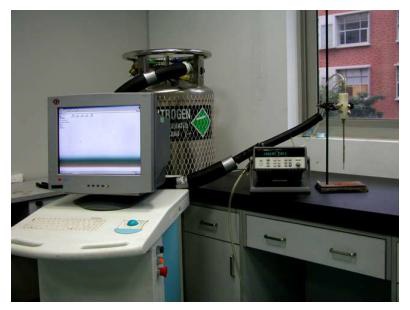


Fig.S2 Actual picture of the ACHT system


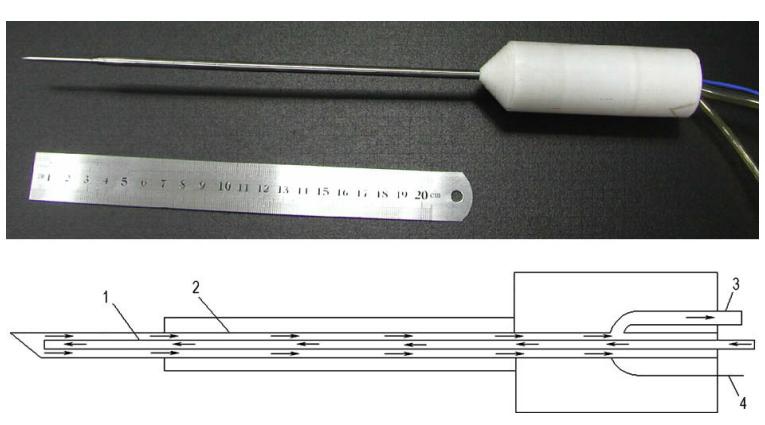


Fig.S3 Structure of ACHT probe[12]

1.Inner tube; 2. Outer tube; 3.Exhaust pipe; 4. Conductor shielded wire
